# Taking promoters out of enhancers in sequence based predictions of tissue-specific mammalian enhancers

**DOI:** 10.1186/s12920-017-0264-3

**Published:** 2017-05-24

**Authors:** Julia Herman-Izycka, Michal Wlasnowolski, Bartek Wilczynski

**Affiliations:** Institute of Informatics, University of Warsaw, Banacha 2, Warsaw, 02-097 Poland

**Keywords:** Enhancer prediction, Regulatory sequence, Histone modifications, Machine learning

## Abstract

**Background:**

Many genetic diseases are caused by mutations in non-coding regions of the genome. These mutations are frequently found in enhancer sequences, causing disruption to the regulatory program of the cell. Enhancers are short regulatory sequences in the non-coding part of the genome that are essential for the proper regulation of transcription. While the experimental methods for identification of such sequences are improving every year, our understanding of the rules behind the enhancer activity has not progressed much in the last decade. This is especially true in case of tissue-specific enhancers, where there are clear problems in predicting specificity of enhancer activity.

**Results:**

We show a random-forest based machine learning approach capable of matching the performance of the current state-of-the-art methods for enhancer prediction. Then we show that it is, similarly to other published methods, frequently cross-predicting enhancers as active in different tissues, making it less useful for predicting tissue specific activity. Then we proceed to show that the problem is related to the fact that the enhancer predicting models exhibit a bias towards predicting gene promoters as active enhancers. Then we show that using a two-step classifier can lead to lower cross-prediction between tissues.

**Conclusions:**

We provide whole-genome predictions of human heart and brain enhancers obtained with two-step classifier.

**Electronic supplementary material:**

The online version of this article (doi:10.1186/s12920-017-0264-3) contains supplementary material, which is available to authorized users.

## Background

Transcription regulation is a complex process requiring tight control at multiple steps including transcription initiation, elongation and splicing. In case of tissue specific genes in metazoan genomes, the control of the transcription initiation is performed largely by means of enhancers, i.e. distinct sequence elements, that allow for binding of transcription factor proteins, facilitating transcription [1]. While the exact molecular mechanism of enhancer-promoter interaction remains a field of active study, we have now accumulated a large body of examples of enhancer sequences to ask whether we can make predictions regarding enhancer location in the genome based on sequence features. This is an important question, given the complexity of a gene regulation system in a multi-cellular organism such as humans composed of hundreds of cell types, each of which expresses thousands of genes, most of which are modulated with some cell-type specificity. Moreover, a typical cell-type-specific gene can be controlled by multiple enhancers. Adding it all up, in order to describe a tissue-specific gene regulation, we need to describe on the order of 100 thousands of regulator elements [2].

Mapping all these elements using experimental techniques is currently completely unfeasible, as many cell-types are too difficult to obtain in large quantities required for experimental assessment of enhancer activity. This leads to a situation, where we have hundreds of well documented examples of regulatory elements functional in a certain context (i.e. cell-type, developmental time) determined by a certain method (enhancer reporter assays [3], STARR-Seq [4], luciferase assays, in-situ hybridization etc). This, however, cannot easily be scaled up to the level of complete coverage of all cell types and all developmental time points. On the other hand, the data collected by the ENCODE or Epigenome Roadmap [5] are invaluable as a source for computational attempts at making models that would be predictive beyond the collected data and perhaps eventually help defining the principles of tissue-specific action of regulatory elements.

Even before the complete sequence of the human genome was known, people have been working on computational descriptions of enhancer sequences – mostly based on clusters of transcription factor binding sites [6, 7]. Later, these models were improved by including evolutionary conservation of sequence features [8] leading to a classical now approach of predicting enhancers as evolutionarily conserved clusters of transcription factor binding sites. While a number of methods following this theme, but varying greatly on the technical side [9–11] has been proposed in the first decade of 2000s, their relative performance was inherently limited by the imperfect training data [12, 13]. The limitations were not only due to the small training sets but also the difficulty to assess the true quality of predictions.

Importantly, while the databases containing the position weight matrices describing transcription factor binding specificity grew rapidly, it became clear that they are not necessarily the best representations of the important sequence features for enhancer predictions [14]. This is due to two main reasons: large similarity of many transcription factor’s binding domains, leading to very similar DNA specificity and the frequently artificial specificity encoded in the position weight matrices based on context-specific determination of binding. Taking the two together, the sequence motif databases were not optimal for the task and it has been shown that the same or better accuracy in enhancer prediction can be achieved with counting k-mers, instead of the actual transcription factor motifs [15].

The advent of the ChIP-Seq technology [16], allowing researchers to directly assay transcription factor binding as well as multiple histone modifications, changed the situation. The availability of genome-wide measurements of transcription factor binding enabled much more comprehensive training of the predictors based on generic machine learning methods [17, 18], however they uncovered an unanticipated complexity of enhancer activity. In particular, the ChIP-Seq based methods allowed us to uncover many regulatory elements that were clearly functional without a detectable sequence conservation between species [17], and the studies of transcription factor binding across developmental time-points or conditions detected large scale context dependency of enhancer function [19]. These findings were later corroborated by multiple studies using DNAse-Seq methods [20, 21].

The wide adoption of ChIP-Seq technique together with concentrated experimental efforts of ENCODE and similar allowed for the new wave of computational approaches to appear. Typically these would take advantage of hundreds genome-wide tracks of ChIP-Seq, DNAse-Seq and other information (such as mRNA-Seq or GRO-Seq) and use a state-of-the-art machine learning method such as SVM [22, 23], Bayesian Networks [18], random forests [24, 25], neural networks [26] or regression based, such as support vector regression [27] or logistic regression [28]. The first study by Erwin et al. should be noted especially for their careful analysis of the specificity of the classifiers. They observed, that when they trained their models on positive vs. random data sets, the resulting classifiers gave overlapping predictions between tissues. Erwin et al. used a specific way of two-layer classification, where they first predicted whether a sequence is an enhancer and in the second step they actually predicted to which class from the training set it belongs. This is an important observation, however, the solution leaves room for improvement as it yields classifiers that are only capable of discerning between the activity classes known at the time of learning. In particular, using this approach one cannot make any claims regarding the overlap of predictions with the regions with activity in any other tissue.

It should be noted that many of these studies differ significantly also in their choice of the training sets. For example Zhu et al. [28] use eRNAs as their positive set, Danko et al. [27] utilize GRO-Seq data, Firpi et al. [26] use histone modification Chip signals preprocessed by Heintzmann et al. [17] while Rajagopal [25] uses p300 distal binding sites. All of these are then very difficult to compare with approaches such as ours or Erwin et al’s [22] that use Vista enhancers.

While enhancer predictions in these studies have reached the level of approximately 90 percent of AUC (Area under the ROC curve) in cross-validated setup, their ability to help us understand the function of enhancers is limited, partially because of their foundation on a very large set of measurements and somewhat opaque machine learning approaches. This has prompted us to approach this problem with a slightly different methodology. We have focused on the issue of enhancer tissue-specificity, i.e. the ability of enhancer sequences to be active only under a very defined set of circumstances and to define a set of features that are crucial for predicting the activity. This has led us to focus less on quality of predictions, but still above “acceptable” level of 80 percent AUC, while retaining the possibility of rigorously assessing the relative importance of the features used for prediction. This has lead to many interesting predictions that are very specific to heart or brain (See Additional file 1: Figure S8, S9 and S10 for representative examples).

In this work we report our findings based on applying Random Forest classification to the problem of enhancer prediction in the human genome. Based on our previous experiences with the *Drosophila* [24, 29], we have used histone modification ChIP-Seq datasets from ENCODE and the enhancer sequences from the Vista project. Using these data, we have defined a set of features that are relevant to define active enhancers and then we went to assess the tissue specificity of the prediction using the heart and brain tissue annotation from the Vista project. This has lead to our discovery that both groups share an enrichment for predictions in the group of proximal-promoter sequences leading to two kinds of problems: firstly both classifiers predict promoters as enhancer and secondly, both classifiers use shared features to predict promoter regions leading to further overlap in genome-wide predictions. In order to tackle this problem we proposed building two-layer classifier, where one layer is responsible for detecting tissue-specific enhancer signals while the other is a pure sequence-based filter of promoter-like sequences that ensures greater specificity of the complete classifier.

## Methods

### Training set and data preparation

**Positive training set** We downloaded all human sequences available in VISTA Enhancer Browser on April 15th, 2014. Our heart training set consists of sequences that show heart activity (among others) but no brain activity, and brain training set is defined as sequences with activity in at least one of those tissues: hindbrain, midbrain, forebrain, neural tube, cranial nerve, but does not show heart activity. Non-specific classifier is trained on both heart and brain sets, defined as above.

**Negative training set** Negative training sets were chosen randomly from the human genome hg19 in the way that they preserve chromosome and length distribution the same as in corresponding positive set. We draw lengths of sequences from Negative Binomial Distribution with parameters matching mean and variance of lengths of sequences in the positive training set. Since heart enhancers in VISTA database are on average longer than brain enhancers (see Additional file 1: Figure S3), we draw separate negative training sets for heart and brain classifiers. We ensure we do not include sequences with ambiguous bases and sequences that have less than 10% of signal in more than quarter of considered histone modifications tracks.

**Sequence features** Our sequence feature set consist of all possible *k*-mers – continuous sequences of *k* bases, excluding second alphabetically *k*-mer for each pair of reverse complement *k*-mers. Each feature is represented by number of occurrences of *k*-mer (or its reverse complement) over length of sequence. Since our random set is chosen in the way, that it doesn’t contain ambiguous bases, we have almost no such bases in the sequences so while counting we skip *k*-mers containing ‘N’.

**Histone modifications features** Histone modification signal was obtained from ChIP-Seq experiment results from ENCODE database. We used downloaded normalized signal for all histone modifications available for cells from Tier1 (H1hESC, GM12878, K562) and normal (non-cancer) cells from Tier2 (CD20+, CD20+_RO01778, CD20+_RO01794, HUVEC, Monocytes-CD14+_RO01746). For list of files see Additional file 1: Table S1. Each ChIP-Seq track contributes one feature to our dataset – mean signal over considered region (e.g. enhancer).

### Training and performance of classifiers

We used Python implementation of random forest from scikit-learn version 0.14.1. We trained initial classifiers with 100 and 1000 trees. Results in terms of AUC were very similar (improvement by 0.02 to 0.16 points between 100 and 1000 trees), so in favor of time we decided to use 100-trees classifier. We used Gini criterion for split quality (default), as well as other default options, which for example ensures building trees that split training samples perfectly.

Each training was performed with positive and negative training sets of equal sizes. If necessary, when positive and negative sets had different size, subset of samples is drawn from larger set before training.

For performance assessment we used stratified 10-fold cross-validation and computed Area Under Receiver-Operator Characteristic Curve (AUC). Presented values are mean AUC from 10 rounds of training (with, when necessary, independently drawn subsets of our training sets).

*P*-value of difference of AUC between two classifiers trained on same training set (but different features) is computed as presented in [30], i.e. it is the *p*-value (two tailed) for a z-score of tested AUC against expected AUC.

### Feature importance

We run Boruta algorithm ([31], version 3.1), a wrapper around Random Forest, that allows assessment of feature importance comparing to random features. It runs multiple rounds of classification (here up to 100) and compares importance of each feature (defined as Z-score of loss of accuracy if feature is permuted), with importance of random features (shuffled initial features). Features that where significantly (*p*-value 0.01, Bonferroni correction), more often more important that best random feature are marked as Important, more often less important that best random feature are marked as Unimportant, and after all of rounds the rest is marked as Tentative.

### Whole-genome predictions

To compute whole-genome predictions we divided hg19 genome (all autosomal chromosomes) into windows of length 1500 bp, every 750 bp. We excluded windows with ambiguous sequence (i.e. containing at least one ‘N’). For each window we output probability of being enhancer – a result of Random Forest voting (as fraction of trees that predict a sequence is active).

### Comparison with DNase hypersensitivity sites

To compare enhancers predictions with DNase hypersensitivity sites we downloaded DNase clusters data (V3) from ENCODE database (https://genome.ucsc.edu/ENCODE/) and DNAse ChIP-Seq data from Roadmap Epigenomics database (http://www.roadmapepigenomics.org/). We compare non-specific 4-mers classifier’s predictions on DHS windows (overlapping DHS clusters by at least 100 bp) with non-DHS windows (other). We excluded windows containing TSS and plot results for 1000 randomly selected DHS and non-DHS windows from the 1-st chromosome.

To define tissue-specific DHS and non-DHS windows we use DNAse ChIP-Seq signal. One thousand windows with highest aggregate signal create DHS set, non-DHS set of 1000 windows is drawn randomly from all windows with maximal signal smaller than 10. We compared distribution of prediction for those sets.

### Promoter predictions and two-step classifiers

We define promoter predictions as those windows from whole-genome 4-mers predictions that have high score (>0.8) and contain at least one TSS from the list of 215,881 TSS from ENSEMBL (downloaded on July 1, 2015). Classifiers trained on promoter predictions are trained on 50 (out of 1775) heart and 50 (out of 632) randomly chosen brain predicted promoters. Second-step classifiers trained on random or VISTA sequences use sequences with length adjusted (extended or shrunken) to 1500 bp, but maintaining same middle position.

### Validation of predictions on new VISTA sequences

After initial training of the classifiers on VISTA sequences few more records were deposited to this database (5 heart and 3 brain enhancers, as of October 10th, 2016). We took the opportunity to validate our classifier on those sequences (see Additional file 1: Table S7). While heart 4-mers classifier predictions were distinguishing heart from brain enhancers (average predictions 0.64 and 0.49), and two-step heart classifier rated heart sequences only slightly above brain (0.3 and 0.27), brain one-step classifier distinguished brain from heart sequences worse (avg. 0.78 and 0.71) than two-step approach (0.52 to 0.33).

## Results

### Predicting mammalian enhancers using random forest classifier

**Fig. 1:**
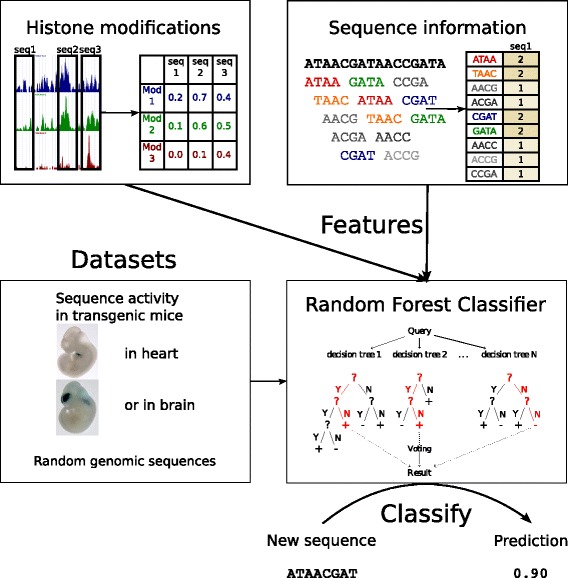
Scheme of our machine learning approach to predict enhancers

Our goal was to use supervised machine learning approach to build a method, that given a set of active enhancers and set of non-enhancers can predict probability of a sequence being active as an enhancer in same tissue as sequences from positive training set. We chose to use Random Forest classifier [32], which performs well on both small and larger feature sets, and enables assessment of importance of individual features. In this method many decision trees trained on subsets of training data are incorporated in order to get a more robust classifier. We trained classifiers on training datasets with various tissue-specificity, aiming to obtain tissue-specific enhancer classifiers (Fig. 1). We also used different feature sets to compare importance of various groups of features for prediction of enhancers.

As our training set we use experimentally validated active enhancers (as positive examples) and random genomic sequences (as a negative set). We took enhancers from VISTA Enhancer Browser [33], database that contains over thousand of human and mouse sequences tested in transgenic mice, with activity confirmed on particular moment of embryonic development. VISTA sequences are also annotated with tissue (or tissues) of activity. For tissue-specific prediction we chose heart and brain tissue, since these where the most abundant in our active enhancers database, and for non-specific we use both heart and brain active sequences.

### Adding histone modifications data to sequence information improves prediction

One of the goals of our work was to check whether random forest classifiers can be effective in combining two types of biological data: DNA sequence of a region and histone modifications within that region to improve prediction accuracy. SVM classifiers were previously shown to do so [22]. As a representation of sequence information we use frequency of *k*-mers — words of length *k*. *k*-mers are simpler model than TFBS motifs and they do not limit information representation only to sequences that represent known TFBS motifs. We took *k*=4, as it gives us relatively small number of features (below 200) while giving slightly better results than 3-mers (see Table 1).

**Table 1:** AUC obtained by different classifiers (mean from 10 rounds of training under 10-fold crossvalidation)

Tissue	Kmers	Hmods	AUC
Non-specific	3 mers	–	0.871
Non-specific	4 mers	–	0.894
Non-specific	4 mers	H1hESC	0.910
Non-specific	4 mers	Tier1&2	0.910
Heart	3 mers	–	0.771
Heart	4mers	–	0.780
Heart	4 mers	H1hESC	0.809
Heart	4 mers	Tier1&2	0.844
Brain	3 mers	–	0.878
Brain	4 mers	–	0.906
Brain	4 mers	H1hESC	0.923
Brain	4 mers	Tier1&2	0.923

Histone modifications data came from ENCODE project ([34]). We used all available at the time ChIP-Seq tracks for histone modifications in the most well studied cell types – cells in Tier 1 (defined by ENCODE as most important to their research cell types) and non-cancer cells from Tier 2 (group with second importance) available at the moment. We will refer to that set as Tier1&2. We also performed training for subset of described data – histone modification only from embryonic stem cells (H1hESC) to see how much of the information from the extensive ENCODE dataset can be extracted from the ground state of the Embryonic stem cells. See list of all used data in Additional file 1: Table S1.

**Fig. 2:**
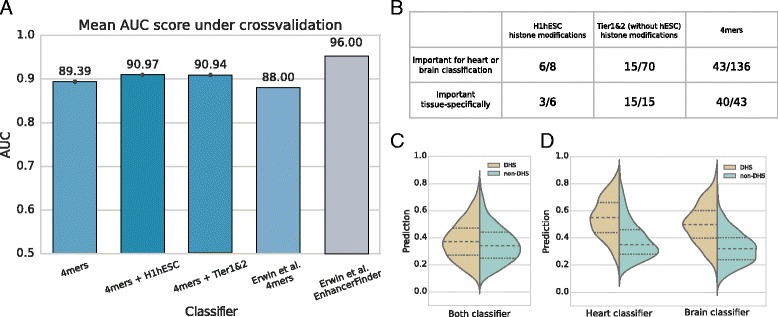
Classifier performance from different perspectives. **a**: Results (as Area Under ROC Curve) of RF classifiers trained on different subsets of training data, compared to EnhancerFinder results, a method developed by Erwin et al., based on SVM and using 4-mers and histone modifications along with TF-binding, DHS and evolutionary conservation. **b**: Number of important features selected with Boruta framework for classifier trained on 4-mers and Tier1&2 histone modifications. *First row* presents how many features where selected important by either heart or brain classifier, whereas *second row* presents how many of those features were important only for one of tissue-specific classifier **c**: Predictions by non-specific (trained on both heart and brain) classifier for genomic windows labeled as non-specific DHS, compared to non-DHS. Scores returned by classifier, can be interpreted as “probability of being enhancer”. **d**: Predictions for tissue-specific (fetal heart and fetal brain) DHS and non-DHS, returned by tissue-specific classifiers

We performed multiple runs of classifier training using different subsets of our feature set and we compared obtained classifiers by measuring their Area Under ROC Curve (AUC). In every case Random Forest classifier gave results comparable with other state of the art methods (AUC between 0.87 and 0.93 — see Table 1). Addition of histone modification data from H1hESC or Tier1&2 improved prediction, although the changes are not significant (*p*-value $\tilde =\ 0.09$, computed as in [30]). More results can be found in the Table 1. These results are strikingly similar to the earlier results by Erwin et al. (see Fig. 2 a) especially when one considers the difference in size of feature sets (besides *k*-mers and evolutionary conservation 2496 features were used for EnhancerFinder training, comparing to 8 for our H1hESC or 78 for Tier1&2 classifier). While the best performance of EnhancerFinder is higher, it is only achieved when it is using evolutionary conservation. However if we do not allow EnhancerFinder to include this data (which is fair, as conservation was part of selection of candidates to the Vista database), its performance is inferior to our method despite much larger number of features.

### Feature importance

Great advantage of Random Forest classifiers over SVM methods is the ability to easily analyse their structure and measure the importance of individual features, e.g. single 4-mers. To do that, we used Boruta algorithm ([31]), a wrapper around Random Forest, that runs classification multiple times and compares feature importance defined as loss of accuracy if feature is permuted, with importance of random features (shuffled initial features). It labels features that are significantly more important than the best random feature as ‘Confirmed’, and rejects the features that are less important than best random. The features that do not meet either of the criteria are labeled ‘Tentative’.

Using Boruta algorithm we found features important for 4 mers+Tier1&2 classifier for heart and brain (see summary in Fig. 2 b, full data in Additional file 1: Table S2). Both types of attributes (sequence and histone modification) were found among important ones: most of histone modifications from H1hESC, some histone modifications from HUVEC and other cell types, and almost one-third of 4-mers. While almost all of the ESC histone features were important for both heart and brain, majority of non-ESC modifications were only predictive in specific tissues. The k-mers were also much more specific in their relevance.

This is consistent with the view that the basic enhancer markers such as H3K4me1 are actually already deposited on enhancers very early in the stem cell stage and the later modifications of these marks are not adding more information for the classifier. However, the early marks may be helpful in finding the negative examples of sequences with the right features, which are positioned in the chromatin context not allowing them to be activated and therefore not marked epigenetically already in the ESC stage. This is consistent with previous reports we found in Drosophila developmental enhancers [18].

### Whole-genome predictions correlate with DHS

Activity of an enhancer is dependent on (among other factors) accessibility of DNA in the region where it is located. DNaseI digestion is one of the main methods used to evaluate this property [21]. Using such measurements, allows us to test our prediction quality on a technically independent experimental dataset, as well as to test (at least to some extent) the true negative predictive value (this is not possible on the randomized training set as we are not sure how many of the random sequences are indeed non-functional. We expect that active enhancers will all be located in open chromatin, although enhancers will consist only part of all DNaseI hypersensitive regions.

We used our classifiers to compute predictions on the whole human genome, divided into overlapping windows of length 1500 bp. We compared values returned by our 4-mers classifiers for windows overlapping DNase Hypersensitive Sites (DHS) and windows without hypersensitivity (non-DHS) (see Fig. 2 c and d). For general set of DHS from the ENCODE [34], derived from multiple cell-lines and not specific to heart or brain, the classifiers results for DHS were slightly, but significantly above results for non-DHS (Mann-Whitney tests *p*-value <10^−4^). Situation was different with fetal heart and brain DNase data from the Epigenomics Roadmap project – our classifier returned clearly higher rates for DHS in compare to specific-non-DHS (*p*-values <10^−130^).

### Tissue-specificity of predictions is low

**Fig. 3:**
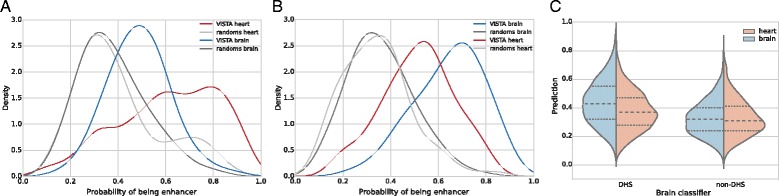
Tissue-specific classifiers trained on 4-mers. **a**, **b**: Distribution of prediction values returned by heart- (**a**) and brain- (**b**) specific classifiers for different sets of sequences. **c**: Distribution of whole-genome prediction values (probability of being enhancer) for DNase-hypersensitive sites (DHS), and regions non-sensitive to DNase (non-DHS)

Even though tissue-specific classifiers seem to work well in predicting heart or brain enhancers against random sequences, they perform worse on enhancers from different tissue (that show now activity in selected tissue), although their confidence is usually lower than those from relevant enhancers (see Fig. 3 a and b). It is also clearly visible in cross-comparison using DNase-Seq data – predictions for DHS windows specific only to heart or brain (windows with promoters or hypersensitive in both tissues excluded) by tissue-specific classifier is small, but significant. For brain classifier see Fig. 3 c (Mann-Whitney test *p*-value <10^−18^ for DHS), for heart classifier Additional file 1: Figure S5) (*p*-value <0.015).

This result is in agreement with the previous reports by Erwin et al., who also noticed significant overlaps in genome wide predictions made by classifiers trained on data from different tissues. While their approach is to use direct machine learning to discern between the known classes of enhancers, we have gone a different route and tried to find a more direct explanation for this problem and a slightly different solution.

### Whole-genome predictions show promoter-bias

**Fig. 4:**
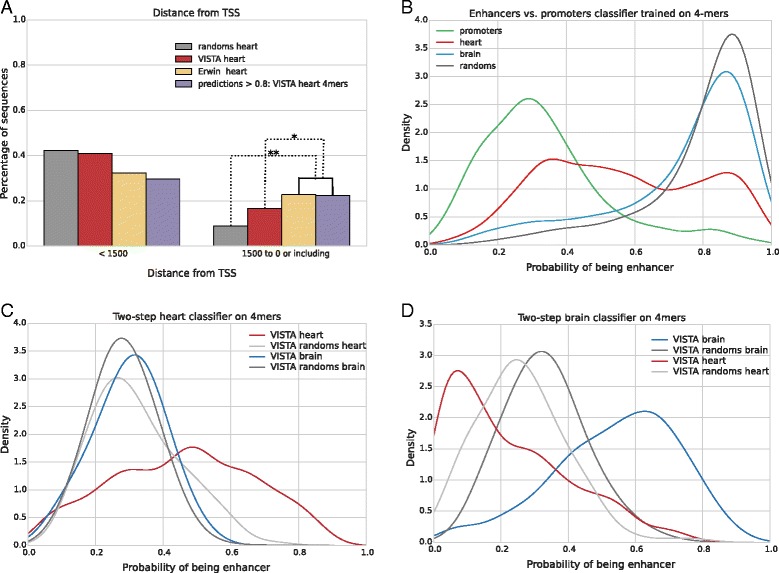
Promoter bias is evident in predicted sequences. **a**: Random Forest classifier, as well as SVM based Erwin et al. predictions, show high preference to promoters. * = *p*-value <10^−100^, ** = *p*-value <10^−250^. **b**: Random Forest can distinguish enhancers (TSS-containing excluded) and promoters (windows containing TSS) with high AUC, using only 4-mers, placing random sequences scores close to enhancers scores. **c** and **d**: Values returned by two-step heart (**c**) and brain (**d**) 4-mers classifier for different sets of sequences

We analyzed locations of high confidence enhancer predictions (i.e. windows with score over 0.8) with respect to nearest transcription start site (TSS) (see Fig. 4 a). We found that out of all 18,376 windows predicted by 4-mers heart classifier 22% were located within 1500 bp upstream of TSS (TSS-proximal), and for 4807 predicted brain enhancers the ratio was 26%.

This is in contrast with our set of random training sequences where promoter proximal sequences amounted to less than 10% (9% of random sequences for heart classifier and 7% of sequences for brain classifier). This is a significant enrichment (binomial test *p*-value <10^−250^). Region of 1500 bases upstream of TSS should contain promoter sequences and many promoter-related transcription factor binding sites, and this promoter-related signal seems to be picked up by the machine learning algorithm leading to a non-specific bias. This problem is not only affecting our method — it is also an issue in EnhancerFinder [22], for which 23% of heart predictions and 16% of brain predictions are TSS-proximal (*p*-value <10^−100^). It is related to the fact that the training sets, especially heart-specific sequences are slightly enriched in regions overlapping promoters (17% for heart, 9% for brain, *p*-values 0.03 and 0.08), however the machine-learning predictions are yet significantly enriched with TSS-proximal regions over the positive examples (*p*-value <10^−80^).

Although promoters are well annotated, so TSS-close predictions can easily be filtered out, we were interested whether we could train our model to discern between enhancers and promoters. If successful, this could help us in the task of predicting tissue-specific enhancer predictors that are not dependent on the knowledge of the “unwanted” enhancer classes, but rather leverage the removal of the promoter bias.

### Two-step classifiers improve specificity

Our first approach was to add enhancers vs. promoters classifiers. As a negative training set we used promoter-containing windows selected by our first-level 4-mers classifiers (heart and brain) with prediction over 0.8. As a positive set we used our previous set of heart and brain enhancers, after adjusting lengths of this sequences to our window length, and removing regions containing TSS. While we were able to discern the enhancers from promoters (AUC = 84%), it proved to be unusable for our purposes as still mixed the promoter and enhancer signatures into a single model. That resulted in the situation, where many random sequences were rated high because of their dissimilarity to the set of promoter sequences (See Fig. 4 b).

For this reason we have turned to training our second classifier on promoter vs. randomly selected sequences. Here we consider random sequences (of length 1500) the positive training set, and promoter windows the negative set. We combine two classifiers by multiplying their predictions, where a high score can be achieved only if the sequence has similar features to the enhancers from the positive set of the first classifier and dissimilar to the promoters of the second classifier. This multiplication of scores leads to slight decrease of the model performance (e.g. AUC from 0.91 to 0.82 for the brain) likely because some of the enhancers indeed include promoter-like features and are likely to be not specific to only one tissue. Nonetheless, we then show that two-step classifiers for both heart and brain indeed show specificity in their prediction against the other class (See Fig. 4 c, d). This is, importantly, despite the fact that the second classifier is not built to specifically exclude the other known tissue, but rather to exclude the non-specific promoter signal.

## Discussion and conclusion

Computational predictions of tissue-specific enhancer activity based on the sequence and epigenetic features is an important field of research, given the complexity of metazoan organisms and the difficulty of obtaining comprehensive experimental measurements of such activity.

Given the current wealth of experimental data, this problem can be formulated as a supervised machine learning task with the positive set taken from an experimental dataset such as the Vista database and the negative set usually taken from a controlled randomized set of genomic sequences.

In our paper, we describe a new approach that uses random forests for this classification task. It has several advantages over the previous studies in this area. In particular, we were able to assess the relative utility of different histone modifications as well as different sequence features for prediction of active enhancers in different tissues.

This allowed us to define a greatly reduced set of features including only histone modifications from the embryonic stem cells (8 ChIP-Seq experiments) and *k*-mers to achieve over 90 percent AUC score.

Using the Boruta package, we were also able to verify which sequence features were the most important and see that the data we have are consistent with the hypothesis that the tissue-specific activity of an enhancer is a combined result of the epigenetic context laid out early in the development and the sequence specific binding of the transcription factors expressed in a given tissue.

We have assessed the genome-wide specificity of such classifiers on the brain and heart related datasets from the Vista database and found that while there is a detectable difference between the positive sequence sets for different tissues, both classifiers are ranking the positive sequences from a different tissue significantly better than the control sequences. This is in agreement with our comparisons with the DNAse-seq data, which point to the same conclusion of the predictions being specific only in comparison with negative controls, but not between classifiers trained on different positive sets.

We found that at least part of this problem is related to the fact that such classifiers are prone to “learning” the enrichment of enhancer-proximal sequences in the positive training sets. This leads to great over-representation of promoter sequences in the predictions of both our classifier as well as the previously published methods.

To find out whether the cause of this observation can be the actual set of sequence features contained in promoter-proximal sequences we have tried to construct a classifier based on purely sequence features that would discern promoters from a control set of sequences. This has proven to be possible and indeed we have further shown that combined classifier using both the promoter related features and the enhancer-trained classifier, is resulting in a much greater specificity between tissues.

We consider our results promising, in the sense that they bring a finer understanding of the mechanisms behind tissue-specific enhancer activity while giving us at the same time useful classifiers and genome-wide predictions. Simultaneously, our results bring new questions into the field. In particular, of our results are correct, we should pay much more attention to the mechanistic difference between enhancers and promoters when building computational methods of analysis and detection of regulatory sequences.

In the long term, these results can lead to better understanding of how mutations in regulatory sequences can disrupt enhancer or promoter function and allow us to better understand the root causes of some genetic diseases.

## Additional file

Additional file 1 Supplementary Tables and Figures. This file contains additional tables and figures, such as table of datasets used for training, feature importance table and predictions for recently added VISTA sequences. (PDF 236 kb)
