# Supplementary material for: Taking promoters out of enhancers in sequence based predictions of tissue-specific mammalian enhancers
Source: BMC Med Genomics. 2017 May 24;10(Suppl 1):34. doi: 10.1186/s12920-017-0264-3 (PMC5461523; doi:10.1186/s12920-017-0264-3)
Supplement: Additional file 1 — Supplementary Tables and Figures. This file contains additional tables and figures, such as table of datasets used for training, feature importance table and predictions for recently added VISTA sequences. (PDF 236 kb) [file 12920_2017_264_MOESM1_ESM.pdf]

Supplementary materials for the paper entitled "Taking promoters out of enhancers in sequence based predictions of tissue-specific mammalian enhancers" by Julia Herman-Izycka, Michal Wlasnowolski and Bartek Wilczynski

Cell type	Modification	Source
H1hesc		ftp://ftp.ebi.ac.uk/pub/databases/ensembl/encode/integration_data_jan2011/byDataType/signal/jan2011/bigwig/
	H3k27ac	wgEncodeBroadHistoneH1hescH3k27acStdAln_2Reps.norm5.rawsignal.bw
	H3k27me3	wgEncodeBroadHistoneH1hescH3k27me3StdAln_2Reps.norm5.rawsignal.bw
	H3k36me3	wgEncodeBroadHistoneH1hescH3k36me3StdAln_2Reps.norm5.rawsignal.bw
	H3k4me1	wgEncodeBroadHistoneH1hescH3k4me1StdAln_2Reps.norm5.rawsignal.bw
	H3k4me2	wgEncodeBroadHistoneH1hescH3k4me2StdAln_2Reps.norm5.rawsignal.bw
	H3k4me3	wgEncodeBroadHistoneH1hescH3k4me3StdAln_2Reps.norm5.rawsignal.bw
	H3k9ac	wgEncodeBroadHistoneH1hescH3k9acStdAln_2Reps.norm5.rawsignal.bw
	H4k20me1	wgEncodeBroadHistoneH1hescH4k20me1StdAln_2Reps.norm5.rawsignal.bw
GM12878		ftp://ftp.ebi.ac.uk/pub/databases/ensembl/encode/integration_data_jan2011/byDataType/signal/jan2011/bigwig/
	H2az	wgEncodeBroadHistoneGm12878H2azStdAln_2Reps.norm5.rawsignal.bw
	H3k27ac	wgEncodeBroadHistoneGm12878H3k27acStdAln_2Reps.norm5.rawsignal.bw
	H3k27me3	wgEncodeBroadHistoneGm12878H3k27me3StdAln_2Reps.norm5.rawsignal.bw
	H3k36me3	wgEncodeBroadHistoneGm12878H3k36me3StdAln_2Reps.norm5.rawsignal.bw
	H3k4me1	wgEncodeBroadHistoneGm12878H3k4me1StdAln_2Reps.norm5.rawsignal.bw
	H3k4me2	wgEncodeBroadHistoneGm12878H3k4me2StdAln_2Reps.norm5.rawsignal.bw
	H3k4me3	wgEncodeBroadHistoneGm12878H3k4me3StdAln_2Reps.norm5.rawsignal.bw
	H3k79me2	wgEncodeBroadHistoneGm12878H3k79me2StdAln_2Reps.norm5.rawsignal.bw
	H3k9ac	wgEncodeBroadHistoneGm12878H3k9acStdAln_2Reps.norm5.rawsignal.bw
	H3k9me3	wgEncodeBroadHistoneGm12878H3k9me3StdAln_3Reps.norm5.rawsignal.bw
	H4k20me1	wgEncodeBroadHistoneGm12878H4k20me1StdAln_2Reps.norm5.rawsignal.bw
K562		ftp://ftp.ebi.ac.uk/pub/databases/ensembl/encode/integration_data_jan2011/byDataType/signal/jan2011/bigwig/
	H2az	wgEncodeBroadHistoneK562H2azStdAln_2Reps.norm5.rawsignal.bw
	H3k27ac	wgEncodeBroadHistoneK562H3k27acStdAln_2Reps.norm5.rawsignal.bw
	H3k27me3	wgEncodeBroadHistoneK562H3k27me3StdAln_2Reps.norm5.rawsignal.bw
	H3k36me3	wgEncodeBroadHistoneK562H3k36me3StdAln_2Reps.norm5.rawsignal.bw
	H3k4me1	wgEncodeBroadHistoneK562H3k4me1StdAln_2Reps.norm5.rawsignal.bw
	H3k4me2	wgEncodeBroadHistoneK562H3k4me2StdAln_2Reps.norm5.rawsignal.bw
	H3k4me3	wgEncodeBroadHistoneK562H3k4me3StdAln_2Reps.norm5.rawsignal.bw
	H3k79me2	wgEncodeBroadHistoneK562H3k79me2StdAln_2Reps.norm5.rawsignal.bw
	H3k9ac	wgEncodeBroadHistoneK562H3k9acStdAln_2Reps.norm5.rawsignal.bw
	H3k9me1	wgEncodeBroadHistoneK562H3k9me1StdAln_1Reps.norm5.rawsignal.bw
	H3k9me3	wgEncodeBroadHistoneK562H3k9me3StdAln_2Reps.norm5.rawsignal.bw
	H4k20me1	wgEncodeBroadHistoneK562H4k20me1StdAln_2Reps.norm5.rawsignal.bw
CD20+		hgdownload.cse.ucsc.edu/goldenPath/hg19/encodeDCC/wgEncodeBroadHistone/
	H2az	wgEncodeBroadHistoneCd20H2azSig.bigWig
	H3k4me2	wgEncodeBroadHistoneCd20H3k04me2Sig.bigWig
	H3k27ac	wgEncodeBroadHistoneCd20ro01794H3k27acSig.bigWig
	H4k20me1	wgEncodeBroadHistoneCd20ro01794H4k20me1Sig.bigWig
CD20+_RO01778		hgdownload.cse.ucsc.edu/goldenPath/hg19/encodeDCC/wgEncodeBroadHistone/
	H3k04me3	wgEncodeUwHistoneCd20ro01778H3k04me3StdRawRep1.bigWig
		wgEncodeUwHistoneCd20ro01778H3k04me3StdRawRep2.bigWig
CD20+_RO01794		hgdownload.cse.ucsc.edu/goldenPath/hg19/encodeDCC/wgEncodeBroadHistone/
	H3k04me3	wgEncodeUwHistoneCd20ro01794H3k04me3StdRawRep3.bigWig
HUVEC		hgdownload.cse.ucsc.edu/goldenPath/hg19/encodeDCC/wgEncodeBroadHistone/
	H2az	wgEncodeBroadHistoneHuvecH2azSig.bigWig
	H3k09me3	wgEncodeBroadHistoneHuvecH3k09me3Sig.bigWig
	H3k27ac	wgEncodeBroadHistoneHuvecH3k27acStdSig.bigWig
	H3k27me3	wgEncodeBroadHistoneHuvecH3k27me3StdSig.bigWig
	H3k36me3	wgEncodeBroadHistoneHuvecH3k36me3StdSig.bigWig
	H3k4me1	wgEncodeBroadHistoneHuvecH3k4me1StdSig.bigWig
	H3k4me2	wgEncodeBroadHistoneHuvecH3k4me2StdSig.bigWig
	H3k4me3	wgEncodeBroadHistoneHuvecH3k4me3StdSig.bigWig
	H3k79me2	wgEncodeBroadHistoneHuvecH3k79me2Sig.bigWig
	H3k9ac	wgEncodeBroadHistoneHuvecH3k9acStdSig.bigWig
	H3k9me1	wgEncodeBroadHistoneHuvecH3k9me1StdSig.bigWig
	H4k20me1	wgEncodeBroadHistoneHuvecH4k20me1StdSig.bigWig
		hgdownload.cse.ucsc.edu/goldenPath/hg19/encodeDCC/wgEncodeUwHistone/
	H3k27me3	wgEncodeUwHistoneHuvecH3k27me3StdRawRep1.bigWig
		wgEncodeUwHistoneHuvecH3k27me3StdRawRep2.bigWig
	H3k36me3	wgEncodeUwHistoneHuvecH3k36me3StdRawRep1.bigWig
		wgEncodeUwHistoneHuvecH3k36me3StdRawRep2.bigWig
	H3k4me3	wgEncodeUwHistoneHuvecH3k4me3StdRawRep1.bigWig
		wgEncodeUwHistoneHuvecH3k4me3StdRawRep2.bigWig
		ftp://ftp.ebi.ac.uk/pub/databases/ensembl/encode/integration_data_jan2011/byDataType/signal/jan2011/bigwig/
	H3k27ac	wgEncodeBroadHistoneHuvecH3k27acStdAln_3Reps.norm5.rawsignal.bw
	H3k27me3	wgEncodeBroadHistoneHuvecH3k27me3StdAln_2Reps.norm5.rawsignal.bw
	H3k36me3	wgEncodeBroadHistoneHuvecH3k36me3StdAln_3Reps.norm5.rawsignal.bw
	H3k4me1	wgEncodeBroadHistoneHuvecH3k4me1StdAln_3Reps.norm5.rawsignal.bw
	H3k4me2	wgEncodeBroadHistoneHuvecH3k4me2StdAln_2Reps.norm5.rawsignal.bw
	H3k4me3	wgEncodeBroadHistoneHuvecH3k4me3StdAln_3Reps.norm5.rawsignal.bw
	H3k9ac	wgEncodeBroadHistoneHuvecH3k9acStdAln_3Reps.norm5.rawsignal.bw
	H3k9me1	wgEncodeBroadHistoneHuvecH3k9me1StdAln_3Reps.norm5.rawsignal.bw
	H4k20me1	wgEncodeBroadHistoneHuvecH4k20me1StdAln_3Reps.norm5.rawsignal.bw
Monocytes-CD14+_RO01746		hgdownload.cse.ucsc.edu/goldenPath/hg19/encodeDCC/wgEncodeBroadHistone/
	H2az	wgEncodeBroadHistoneMonocd14ro1746H2azSig.bigWig
	H3k04me1	wgEncodeBroadHistoneMonocd14ro1746H3k04me1Sig.bigWig
	H3k04me2	wgEncodeBroadHistoneMonocd14ro1746H3k04me2Sig.bigWig
	H3k04me3	wgEncodeBroadHistoneMonocd14ro1746H3k04me3Sig.bigWig
	H3k09ac	wgEncodeBroadHistoneMonocd14ro1746H3k09acSig.bigWig
	H3k09me3	wgEncodeBroadHistoneMonocd14ro1746H3k09me3Sig.bigWig
	H3k27ac	wgEncodeBroadHistoneMonocd14ro1746H3k27acSig.bigWig
	H3k27me3	wgEncodeBroadHistoneMonocd14ro1746H3k27me3Sig.bigWig
	H3k36me3	wgEncodeBroadHistoneMonocd14ro1746H3k36me3Sig.bigWig
	H3k79me2	wgEncodeBroadHistoneMonocd14ro1746H3k79me2Sig.bigWig
	H4k20me1	wgEncodeBroadHistoneMonocd14ro1746H4k20me1Sig.bigWig
		hgdownload.cse.ucsc.edu/goldenPath/hg19/encodeDCC/wgEncodeUwHistone/
	H3k04me3	wgEncodeUwHistoneMonocd14ro1746H3k04me3StdRawRep1.bigWig
	H3k27me3	wgEncodeUwHistoneMonocd14ro1746H3k27me3StdRawRep1.bigWig

Table S1: Histone modification tracks from ENCODE used in analysis.

Tissue	# sequences in VISTA	after filtering
heart	91	78
brain (hindbrain, midbrain, forebrain, neural tube, cranial nerve)	571	558
non-specific (heart + brain)	649	636

Table S2: Training set sizes

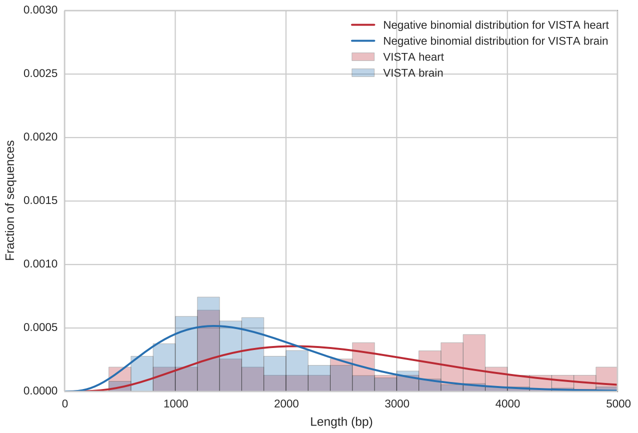

Figure S3: Lengths of sequences from VISTA database with fitted distribution.
Heart (and not brain): mean=2651.74, variance=1525898.65
Brain (and not heart): mean=1770.32, variance=743470.65

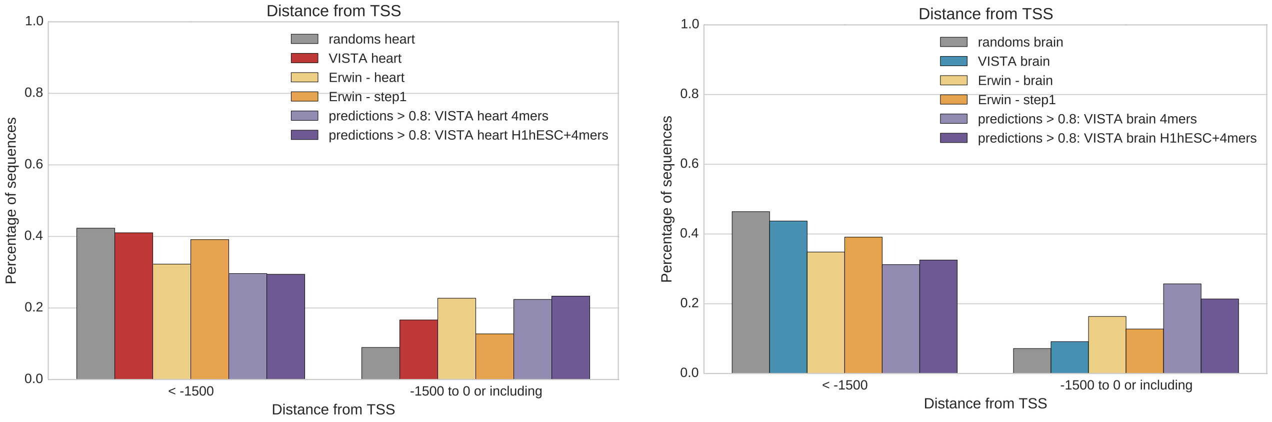

Figure S4: Fraction of sequences in datasets with specific distance to closest TSS.

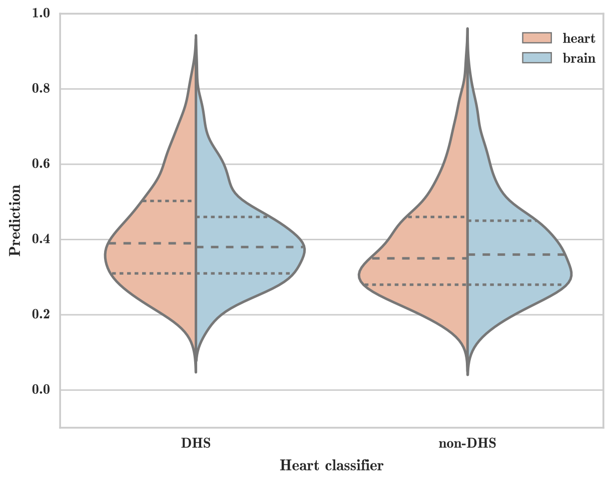

Figure S5: Distribution of whole-genome prediction values for DNase Hypersensitive Sites windows specific to heart and brain (promoters and heart&brain DHS removed) and non-DHS windows.

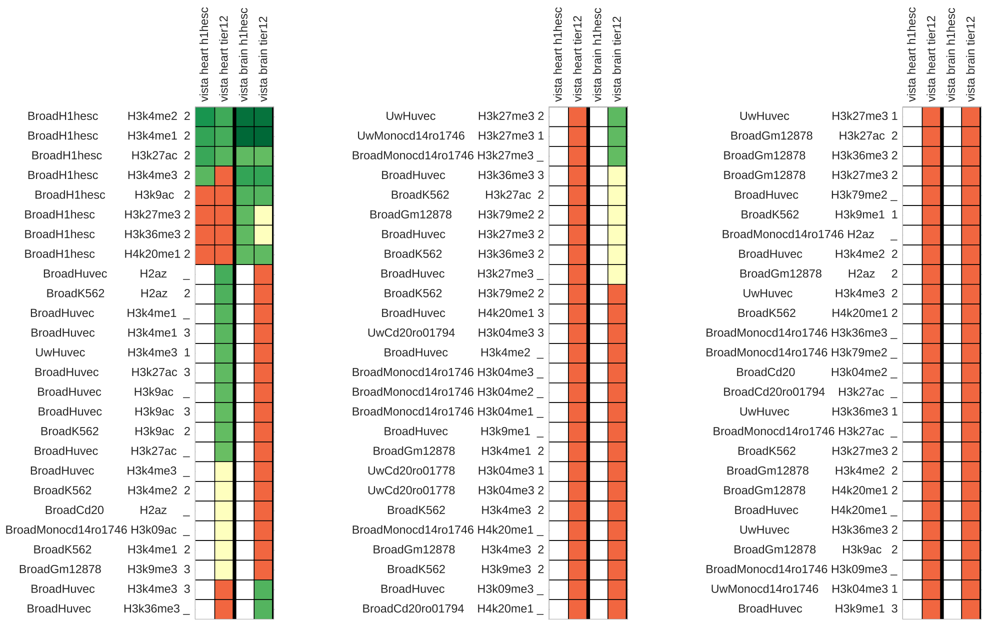

Figure S6: Feature importance by Boruta algorithm
Comparison of important 4-mers (first figure) and histone modifications (second figure) for tissue-specific 4-mers classifiers (without histone modifications, with H1hESC and with Tier1&2). Important features in green, Unimportant in red and Tentative in yellow.

		Classifier							
		heart				brain			
element ID	tissue	4-mers	4-mers + H1hesc	4-mers + Tier1&2	2-step 4-mers	4-mers	4-mers + H1hesc	4-mers + Tier1&2	2-step 4-mers
2315	brain	0.57	0.57	0.39	0.2025	0.96	0.74	0.68	0.4005
2318	brain	0.39	0.38	0.41	0.3026	0.7	0.86	0.76	0.6408
2319	brain	0.5	0.62	0.61	0.315	0.68	0.81	0.79	0.5175
2330	heart	0.61	0.76	0.7	0.495	0.79	0.64	0.5	0.5775
2353	heart	0.6	0.63	0.8	0.304	0.44	0.51	0.54	0.184
2357	heart	0.76	0.7	0.77	0.2272	0.69	0.51	0.56	0.2112
2384	heart	0.75	0.71	0.82	0.1343	0.7	0.39	0.34	0.1207
2414	heart	0.46	0.43	0.45	0.3348	0.92	0.42	0.43	0.5642

Table S7: Predictions for recently added VISTA enhancers given by various classifiers anal- ysed in this paper.

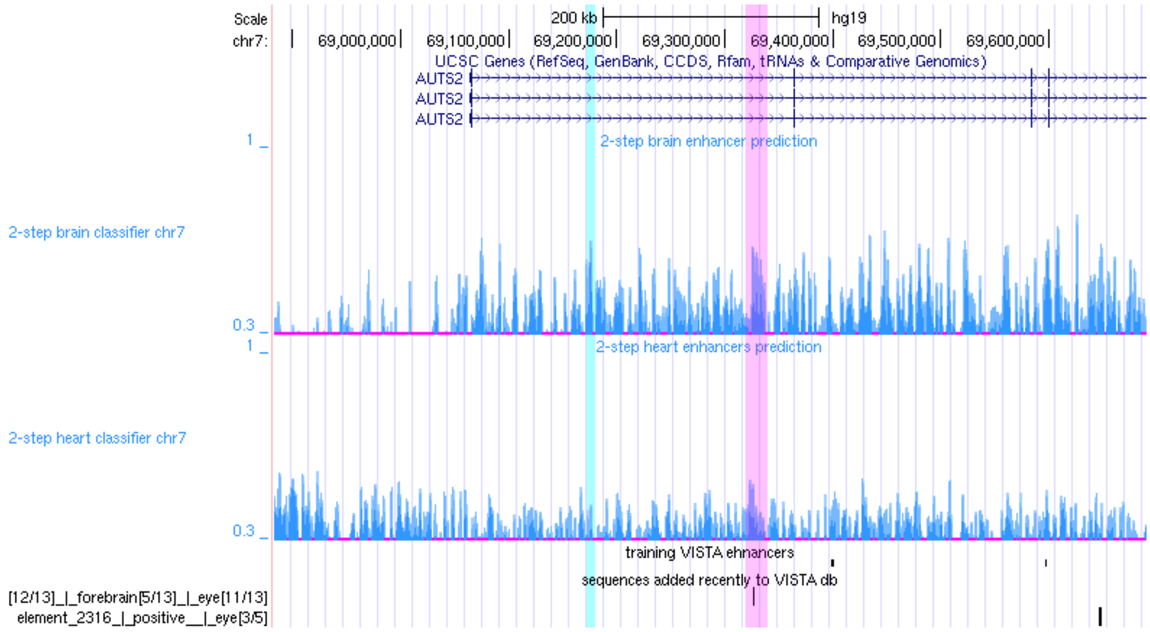

Figure S8: Example of region containing brain enhancer. We highlighted one of regions with high brain prediction, and low heart (in blue). Another such region (in pink) overlaps VISTA brain enhancer, which wasnotpartoftrainingset.BothregionsarelocatedinsideautismrelatedgeneAUTS2.

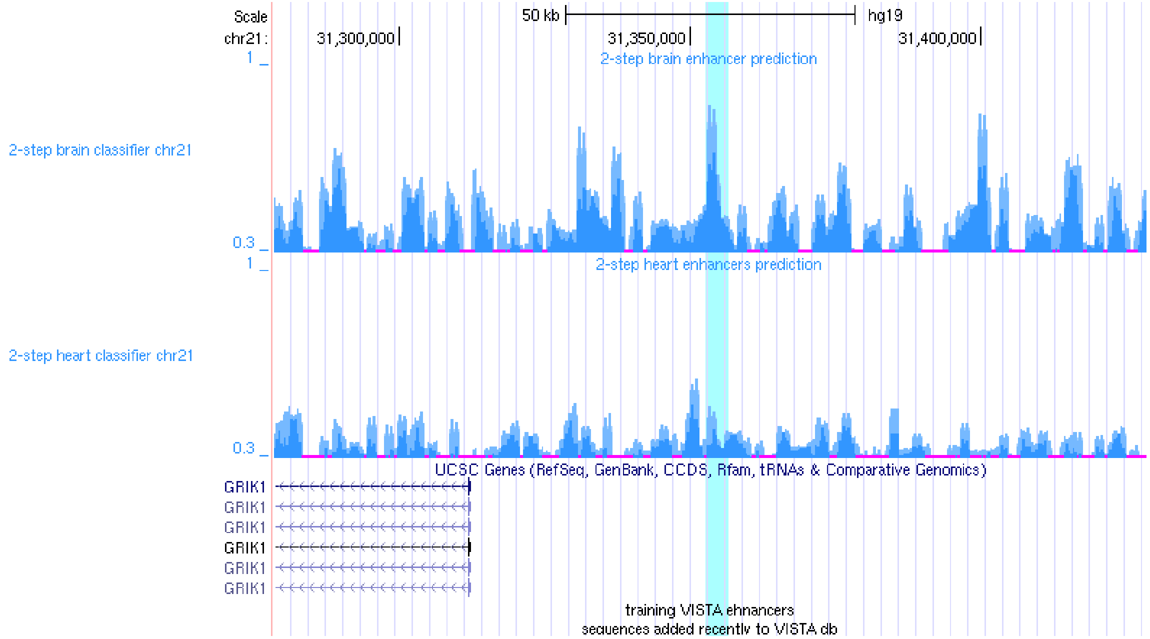

Figure S9: Example of region containing brain enhancer prediction. We highlighted one of regions with highbrainprediction,andlowheart.RegionislocatedinproximityofglutamatereceptorgeneGRIK1.

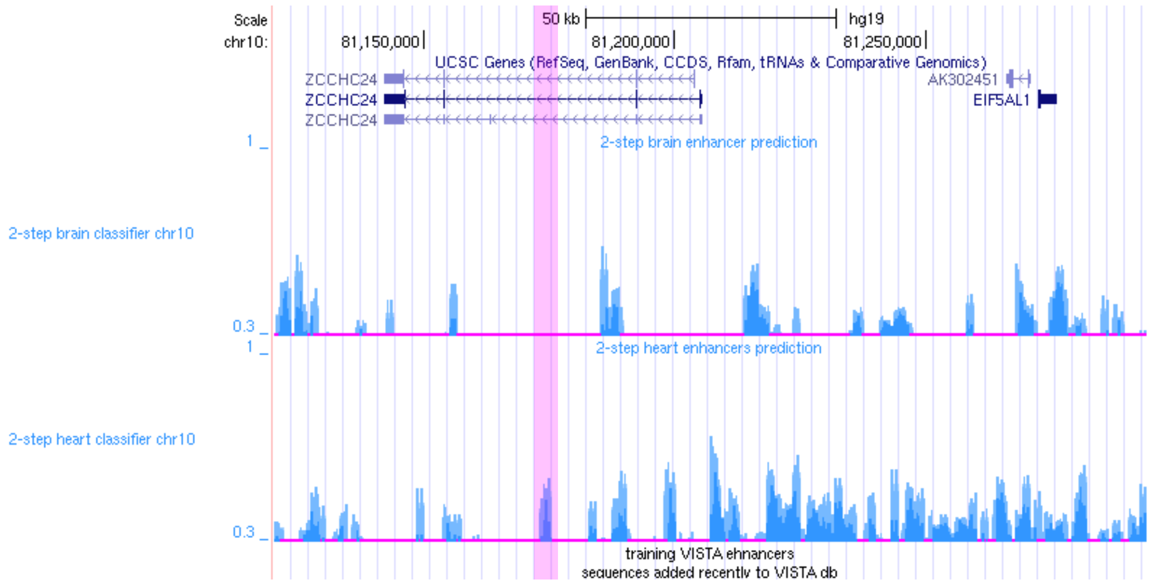

Figure S10: Example of region containing heart enhancer prediction (highlighted in pink) located inside one of introns of gene ZCCHC24 (Zinc Finger CCHC Domain-Containing Protein 24).
